# Understanding the Demographic Predictors and Associated Comorbidities in Children Hospitalized with Conduct Disorder

**DOI:** 10.3390/bs8090080

**Published:** 2018-09-04

**Authors:** Rikinkumar S. Patel, Neelima Amaravadi, Harkeerat Bhullar, Jay Lekireddy, Honey Win

**Affiliations:** 1 Department of Psychiatry, Griffin Memorial Hospital, 900 E Main St, Norman, OK 73071, USA; 2 Department of Pediatrics, Oakleaf Eau Claire Medical Clinic, 3802 Oakwood Mall Dr, Eau Claire, WI 54701, USA; neelimaamaravadi9@gmail.com; 3 Department of Pediatrics, Windsor University School of Medicine, Brighton’s Estate, Cayon, St. Kitts P.O. Box 1621, Saint Kitts and Nevis; kbhullar7@gmail.com; 4 Saint Barnabas Hospital, 4422 Third Ave, Bronx, NY 10457, USA; jlekireddy@gmail.com; 5 Department of Psychiatry, Icahn School of Medicine at Mount Sinai/Elmhurst Hospital Center, 79-01 Broadway, Elmhurst, NY 11373, USA; winh@nychhc.org

**Keywords:** conduct disorder, comorbidities, schizophrenia, demographics, child, behavior, child psychiatry

## Abstract

**Objective:** To determine the demographic predictors and comorbidities in hospitalized children with conduct disorder. **Methods:** A retrospective analysis was performed using the Nationwide Inpatient Sample (2012–2014). All patients were ≤18 years old and cases with a primary diagnosis of conduct disorder (*n* = 32,345), and a comparison group with another psychiatric diagnosis (*n* = 410,479) were identified using the International Classification of Diseases, Ninth Revision, Clinical Modification (ICD-9-CM)diagnosis codes. A logistic regression model was used to generate the odds ratio (OR) between both groups. **Results:** Children < 11 years old have a five times greater chance of admission for conduct disorder than adolescents (OR = 5.339). African American males are more likely to be admitted for conduct disorder. Children with conduct disorder from low-income families have a 1.5 times higher likelihood of inpatient admission compared to high-income families. These children have an about eleven times higher odds of comorbid psychosis (OR = 11.810) and seven times higher odds of depression (OR = 7.093) compared to the comparison group. **Conclusion:** Conduct disorders are more debilitating for children and families than many providers realize. African American males under 11 years are at the highest risk of inpatient management for conduct disorder. These patients have a higher risk of comorbid psychosis and depression, which may further deteriorate the severity of illness and require acute inpatient care.

## 1. Introduction

Conduct disorder is defined as a repetitive and persistent pattern of behavior in which the basic rights of others or major age-appropriate societal norms or rules are violated [1]. Children or adolescents with conduct disorder exhibits aggressive behavior, such as bullying, threatening, initiating physical fights, cruelty towards animals, destruction of property, stealing and serious violations of rules in a variety of settings [1]. Conduct disorder symptoms are the most common primary presenting problems for psychiatric referral among children and adolescents in the United States [2], and youth diagnosed with conduct disorder have a higher degree of distress and impairment in virtually all domains of living than youth with other mental disorders [2,3]. The estimated lifetime prevalence of conduct disorder in the United States is 9.5% (12% in males and 7.1% in females) with median age of onset of 11.6 years [2]. An epidemiological meta-analysis estimated that the worldwide prevalence of conduct disorder among children and adolescents aged 6–18 years is 3.2% and the prevalence estimate does not vary significantly across countries [4].

Conduct disorder can have its onset before ten years of age or in adolescence, and children with early-onset conduct disorder are at greater risk for persistent difficulties. Current data indicates that the prevalence of conduct disorder is 2–5% in children between 5–12 years and 5–9% in adolescents between 13–18 years [5]. Most studies show that boys are more likely to present with symptoms of conduct disorder than girls. However, this gender difference may vary somewhat across development. In young children under five years age, gender differences are small [6]. This changes in adolescence, where both genders show an increase in the rates of conduct disorder and boys are two to three times more likely to be diagnosed than girls [7]. Conduct disorder prevalence may or may not vary in different races and ethnicities depending on socioeconomic status, neighborhood and parenting practices. According to current data, the lifetime prevalence of conduct disorder is 6.9% in Hispanics, 4.9% in Blacks and 5.0% in Whites [8]. Caucasian children are more likely to be diagnosed with oppositional defiant disorder, whereas African American children are more likely to be diagnosed with conduct disorder [9]. Male teens, minorities and children from low-income families are likely to be diagnosed with severe problems linked to neurological, attention, and conduct functioning [9].

Many studies have demonstrated the long-term impact of conduct disorder as a developmental precursor of later antisocial behavior and criminality [10,11,12,13]. Conduct disorder diagnosed in childhood acts as a strong predictor of many problems in adolescence and adulthood, including mental illness, substance abuse, legal problems, school drop-out and academic issues and occupational problems [14,15]. In two longitudinal studies, children with comorbid conduct and depressive disorders had a higher risk of late-onset criminality and antisocial behavior compared to those with only emotional issues [16,17]. Moreover, studies in the past have shown that conduct problems are associated with an increased risk of other mental disorders [12]. Many children with a conduct disorder may have coexisting conditions such as ADHD (3–41%), depression (0–46%) and anxiety disorder (0–41%) [18]. A birth cohort study in New Zealand showed that boys who had a conduct disorder prior to adolescence were three times more likely to have an anxiety disorder and major depressive disorder, eight times more likely to be homeless, three times more likely to be dependent on alcohol, and 25 times more likely to have attempted suicide by age 32 years compared to boys without a conduct disorder [14].

Despite the significant impact of conduct disorder on society, the economy, and healthcare, no prior studies have been done to determine demographic differences in the inpatient population with conduct disorder compared to other psychiatric disorders in children and adolescent populations in the United States. There are questions regarding the widely documented comorbidity of conduct disorder with other mental disorders [12]. Although it is clear that conduct disorder is associated with other disorders, little is known about the prevalence of these comorbid disorders, specifically, very little data is available regarding the likeliness of having schizophrenia and psychotic disorders, depression, and substance/alcohol use disorders as a comorbidity with conduct disorder. Thus, the purpose of this study is to determine the demographic predictors and association of comorbidities in children hospitalized with a primary psychiatric diagnosis of conduct disorder.

## 2. Methods

### 2.1. Data Source

A retrospective analysis was performed using the Healthcare Cost and Utilization Project’s (HCUP) Nationwide Inpatient Sample (NIS) data from the years 2012 to 2014 [19]. The Agency for Healthcare Research and Quality (AHRQ) sponsors the HCUP databases that are specifically designed to determine and identify patterns in hospital utilization and cost across the United States. The HCUP-NIS database is the largest inpatient database available in the United States that represents a sample of non-federal United States community hospitals [19].

### 2.2. Variables of Interest

Based on the International Classification of Diseases, Ninth Revision, Clinical Modification (ICD-9-CM) diagnosis codes, we identified the patients with a primary diagnosis of conduct disorder at the time of admission as the target group, and the comparison group with a primary psychiatric diagnosis other than conduct disorder at the time of admission. The target group included the patients who were primarily managed for conduct disorder only, and thus those patients with a primary diagnosis of oppositional defiant disorder were excluded. The patients in the comparison group were excluded if they had a secondary or other diagnosis of conduct disorder. In the HCUP databases, more than 14,000 ICD-9-CM diagnosis codes and 3,900 procedure codes were recorded [19].

Patients in the comparison group were identified using the diagnosis codes for anxiety disorder (293.84, 300.00–300.02, 300.09, 300.10, 300.20–300.23, 300.29, 300.3, 300.5, 300.89, 300.9, 308.0–308.4, 308.9, 309.81, 313.0, 313.1, 313.21, 313.22, 313.3, 313.82, 313.83), mood disorder (293.83, 296.00–296.06, 296.10–296.16, 296.40–296.46, 296.60–296.66, 296.7, 296.50–296.56, 296.82, 296.80, 296.89, 296.7, 296.20–296.26, 296.30–296.36, 296.82, 300.4, 311, 293.83, 296.90 or 296.99) and schizophrenia and other psychotic disorders (293.81, 293.82, 295.00–295.05, 295.10–295.15, 295.20–295.25, 295.30–295.35, 295.40–295.45, 295.50–295.55, 295.60–295.65, 295.70–295.75, 295.80–295.85, 295.90–295.95, 297.0–297.3, 297.8–298.4, 298.8 or 298.9). Patients with conduct disorder were identified using the diagnosis codes 312.00–312.03, 312.10–312.13, 312.20–312.23, 312.4, 312.8, 312.81, 312.82, 312.89 and 312.9. The age limit was set ≤18 years to compare the hospitalization outcomes and comorbidities between conduct disorder and other psychiatric disorders in children and adolescents.

The demographic variables examined in this study included age group (<11 years, 12–18 years), gender (male or female), race (Caucasian, African American, Hispanic and other) and median household income (below/above 50th percentile). Comorbidities were considered coexisting conditions to conduct disorder, which was the primary disorder in this study [20]. Using ICD-9-CM codes, this variable identified four comorbidities in the records of patient during hospitalization—alcohol abuse (291.0–291.3, 291.5, 291.8, 291.81, 281.82, 291.89, 291.9, 303.00–303.93, 305.00–305.03), drug abuse (292.0, 292.82–292.89, 292.9, 304.00–304.93, 305.20–305.93, 648.30–648.34), psychosis (295.00–298.9, 299.10, 299.11), and depression (300.4, 301.12, 309.00, 309.1, 311).

### 2.3. Approaches

A retrospective analysis was performed of the HCUP-NIS database, focusing on the determination of the hospital outcomes for conduct disorder patients and controls. Descriptive statistics were used to summarize the results. The mean and standard deviations were used to explain the continuous variables. The Pearson’s chi-square test and independent sample *t*-test were used for categorical data and continuous data, respectively. We used a multinomial logistic regression model to measure the risk of associations (odds ratio (OR)) between both groups in terms of demographic predictors and comorbidities. Separate regression models were used to evaluate the demographic predictors in the target group. To evaluate the odds of comorbidities like alcohol abuse, drug abuse, depression and psychosis, separate regression models were analyzed with comorbidity (yes or no) and covariates (age, gender and race) with the comparison group of patients without conduct disorder as the reference category. We applied discharge weights in all the regression models to obtain nationally representative inpatient data. A *p* value < 0.01 was used as a reference to determine the statistical significance of the test result. All statistical analysis was done using SPSS 23 in this study [21].

### 2.4. Ethical Approval

Our database did not contain patient personal identifying information. To protect the privacy of individual patients, physicians, and hospitals, the state and hospital identifiers were de-identified. Thus, we were not required to obtain institution review board permission for this study.

## 3. Results

### 3.1. Sample Characteristics

The study analyzed a total of 442,824 children admitted to hospital for primary psychiatric diagnosis from 2012 to 2014. The comparison group (*n* = 410,479) included children admitted with a primary diagnosis of anxiety disorder (5.1%), mood disorder (3.3%) and schizophrenia or other psychotic disorders (91.6%). The target group included 32,345 children admitted in the hospital for conduct disorder.

### 3.2. Demographic Differences

About 55% of the children admitted to hospital for conduct disorder were adolescents (12–18 years) and 70% were male. The mean age of the comparison group was 14.4 years (±2.827), which was higher than the patients with conduct disorder, with an average of 11.6 years (±3.606). Only 13% of the children in the comparison group were under 11 years, compared to 44% of those with conduct disorder. Thus, children in the <11 years age group have a five times greater chances of admission for conduct disorder than adolescent (OR 5.339). Males are three times more likely to be admitted for conduct disorder than females (OR 3.339). The results of the racial sub-group analysis found that the majority of the patients admitted for conduct disorder were African American (29.4% vs. 16.7%) and Hispanic (15.2% vs. 13.2%) when compared with the comparison group. African Americans were two-fold more likely to be admitted for conduct disorder than other races.

The majority of the patients with conduct disorder were covered by Medicaid (65%), followed by private insurance (29.4%). Children with conduct disorder from low-income families with a median household income below the 50th percentile have a 1.5 times higher likelihood of inpatient admission than those from high-income families. The demographic distribution of the study population is shown in Table 1.

### 3.3. Association of Comorbidities

The most prevalent comorbidities in conduct disorder patients were psychosis (29%) followed by depression (14.9%), and the least common was drug abuse (6%). Children with conduct disorder had a lower likelihood of comorbid alcohol abuse and drug abuse compared to other children admitted with other psychiatric diagnoses. These children with conduct disorder have an about eleven times higher odds of comorbid psychosis (OR = 11.810) and a seven times higher odds of depression (OR = 7.093) compared to the comparison group, as shown in Table 2.

## 4. Discussion

This study included 442,824 children ≤18 years old who were admitted to hospital with a psychiatric disorder; 32,345 had an admitting diagnosis of conduct disorder. As per a retrospective study conducted on in the National Comorbidity Survey Replication (NCS-R), the estimated prevalence of conduct disorder in the United States is 9.5%, with a median age of onset of 11.6 years [2]. A meta-analysis that included studies from 1987 to 2008 concluded that the worldwide prevalence of conduct disorder among children aged 6–18 years is 3.2% and only methodological and not geographic factors were related to variability of the prevalence [4]. None of the studies estimated the nationwide inpatient prevalence of conduct disorder, and our results show that about 7.3% of children with mean age of 11.6 years of total inpatient psychiatric admissions had a primary diagnosis of conduct disorder. Children under 11 years have a five times greater chance of inpatient admission for conduct disorder than adolescents.

About 70% of the children with conduct disorder in the current study were males, and they were three times more likely to be admitted for conduct disorder than females. Our results are supported by a longitudinal study conducted by Moffitt et al. [7] that concluded that boys are two to three times more likely to be diagnosed with conduct disorder than girls. A study of the National Comorbidity Survey Replication stated that conduct disorder prevalence may or may not vary according to different races/ethnicities and socioeconomic status [8]. However, in our study we were able to discern a racial pattern, as the majority of the patients admitted for conduct disorder were Caucasians, but in our regression model we found that African Americans had a two-fold higher risk of being admitted for conduct disorder. A study by Proctor et al. [9] stated that African American children are more likely to be diagnosed with conduct disorder. Also, children from low-income families have serious concerns in terms of conduct and attention functioning [9]. In our study’s regression analysis, we found that children from families with a median household income below the 50th percentile had a 1.5-fold higher odds of psychiatric hospitalization for conduct disorder. Previous studies have stated that low income families with concerning surroundings are predominant among children with conduct disorders [22,23]. D’Onofrio et al. [24] appealed that there is a causal association between family income and conduct disorder, and emphasized the importance of recognizing household family income as a critical risk factor for the development of early-onset conduct disorder.

A birth cohort study in New Zealand showed that boys who had conduct disorder were three times more likely to have an anxiety disorder and major depressive disorder, and be dependent on alcohol compared to boys without conduct disorder [14]. The conduct disorder cohort in our study had a seven times higher odds of depression, but were less likely to have a co-diagnosis of drug abuse and alcohol abuse. The strongest association between conduct disorder and substance use disorder emerged at age 15 for both males and females [25]. A past history of conduct disorder had a robust additive effect at age 16, when substance use and abuse became normative [26]. However, there is a considerable heterogeneity among youth with eminent symptoms of conduct disorder, and early internalizing problems like depression and anxiety that lead to late-onset alcohol use disorder [25,27,28]. The children with conduct disorder in our study were younger (mean age 11.6 years), which could be the main reason for a lower association with substance use disorder compared to children with other psychiatric illnesses. Many studies in the past have shown an association of schizophrenia and conduct disorder [29,30,31,32,33], and we also found an about eleven-fold higher likelihood of comorbid schizophrenia and other psychotic disorders in children admitted for conduct disorder. 

The key strength of this study is the national representation provided by the NIS dataset, with a uniform collection of data using ICD-9-CM codes, and its large sample size of 442,824 children under 18 years of age. This study includes children with a psychiatric diagnosis of conduct disorder versus those without conduct disorder. The identification of patients using the ICD-9-CM diagnosis code may be affected by some external factors such as insurance and billing. We were not able to evaluate the odds of other psychiatric comorbidities in the study population. The clinical and non-clinical information in this dataset are coded independently of the individual practitioner and thus it is subjected to minimal reporting bias. Nevertheless, our study does have few limitations as it is an administrative dataset. Re-hospitalizations, which add to the total inpatient burden, were not accounted for in our study. However, despite these limitations, NIS is still an excellent population-based representation of disease associations with comorbidities.

## 5. Conclusions

Conduct disorders are more debilitating for children and families than many providers realize. African American males under 11 years are at the highest risk of inpatient management for conduct disorder. These patients have a higher risk of comorbid psychosis and depression, which may further deteriorate the severity of illness and require acute inpatient care. The cultural impact on parenting behavior is often seen in African American families, as parents are more likely to apply physical punishment and emotional withdrawal than other races/ethnicities, since they place value on obedience [34]. Thus they teach pain and coping skills to their children and prepare them to tackle pain and disappointment instead of being protected from such factors [35]. Morgan et al. found that African American parents of low socio-economic status mostly teach their children how to survive (like coping with racism) rather than teaching quiet behaviors [36]. Further studies should be done to highlight the growing issue of conduct disorder and the necessity to develop biopsychosocial care models for prompt diagnosis and treatment.

## Figures and Tables

**Table 1:** Demographic predictors of hospitalized patients with conduct disorder.

Characteristic	CD (−)		CD (+)		Logistic Regression Model		
	*n*	%	*n*	%	OR	95% CI	*p* Value
**Age**							
<11 years	53,695	13.1	14,410	44.6	5.339	5.214–5.467	<0.001
12–18 years	356,784	86.9	17,935	55.4	Referent		
**Gender**							
Male	166,054	40.5	22,570	69.8	3.339	3.316–3.483	<0.001
Female	244,414	59.5	9775	30.2	Referent		
**Race**							
Caucasian	227,555	62.9	13,255	48.4	0.794	0.756–0.834	<0.001
African American	60,475	16.7	8055	29.4	1.816	1.724–1.912	<0.001
Hispanic	47,805	13.2	4180	15.2	1.192	1.127–1.261	<0.001
Other	26,175	7.2	1920	7.0	Referent		
**Primary payer**							
Medicaid	193,574	47.3	21,050	65.5	2.294	2.145–2.453	<0.001
Private insurance	185,870	45.4	9440	29.4	1.459	1.321–1.613	0.049
Self-pay	10,480	2.6	725	2.3	1.071	1.000–1.148	<0.001
Other	19,725	4.8	935	2.9	Referent		
**Median household income**							
0–50th percentile	214,624	53.4	19,515	64.0	1.553	1.516–1.591	<0.001
51st–100th percentile	187,325	46.6	10,970	36.0	Referent		

Differences between groups conducted by cross-tabulation. Components may not add up to the rounded sum due to weighting and rounding or missing data. The odds ratio generated using the binary logistic regression model and the reference category for this model are mentioned within the table. CD: conduct disorder; OR: odds ratio; CI: confidence interval.

**Table 2:** Association of comorbidities in patients with conduct disorder.

Comorbidity	CD (−)		CD (+)		Logistic Regression Model		
	*n*	%	*n*	%	OR	95% CI	*p* Value
**Alcohol abuse**	75,705	18.4	4195	13.0	0.700	0.672–0.729	<0.001
**Drug abuse**	24,470	6.0	1250	3.9	0.758	0.706–0.814	<0.001
**Depression**	11,245	2.7	4825	14.9	7.093	6.781–7.420	<0.001
**Psychosis**	12,950	3.2	9365	29.0	11.810	11.399–12.235	<0.001

Differences between groups conducted by cross-tabulation. Components may not add up to the rounded sum due to weighting and rounding or missing data. The odds ratio was generated using the binary logistic regression model and was adjusted for age, gender, and race and median-household income. The reference category for this model was patients without conduct disorder (comparison group). CD: conduct disorder; OR: odds ratio; CI: confidence interval.
